# The Retrourethral Transobturator Sling Suspension in the Treatment of Male Urinary Stress Incontinence: Results of a Single Institution Experience

**DOI:** 10.5402/2012/304205

**Published:** 2012-05-17

**Authors:** Johannes Mueller, Andres Jan Schrader, Thomas Schnoeller, Friedemann Zengerling, Ilija Damjanoski, Andreas Al Ghazal, Mark Schrader, Florian Jentzmik

**Affiliations:** Department of Urology, University Hospital Ulm, Prittwitzstrasse 43, D-89075 Ulm, Germany

## Abstract

*Objective*. To evaluate functional outcome of the retrourethral transobturator sling suspension (RTS) in the treatment of stress urinary incontinence (SUI) caused by prior prostate surgery. *Methods*. The RTS (AdVance male sling) was implanted in 32 patients who suffered from mild to severe postsurgical-treatment incontinence at the University Hospital Ulm from September 2010 to September 2011 including 10 patients with prior radiation therapy. Functional data (uroflowmetry, daily pad use, and postvoid residual urine) as well as quality of life with impact of urinary problems (ICIQ-UI SF) were prospectively assessed at baseline and during followup. *Results*. After a median followup of 9 months (range, 3–14) the incontinence cure rate (no pad usage) was 56.2% and the improvement rate (1-2 pads/day or ≥50% reduction) was 21.9%. No improvement was observed in 21.9%. Daily pad use and ICIQ-UI SF score improved significantly. No major perioperative complications occurred. Postoperatively, 15.6% of the patients exhibited transient acute urinary retention which resolved without further treatment after a maximum of 3 weeks. One patient underwent sling explantation due to dislocation and persistent perineal pain. *Conclusions*. The implantation of the RTS is a safe and effective procedure in selected patients with SUI resulting from prostate surgery.

## 1. Introduction

Radical prostatectomy is regarded as the gold standard surgical treatment for organ confined prostate cancer. Even though the surgical technique has been improved steadily stress urinary incontinence is a well-known side effect of this procedure with reported incidence rates of up to 20% [1, 2]. Another rare reason for postsurgery incontinence is the transurethral resection (TUR) of the prostate [3]. The artificial urinary sphincter is still considered to be the standard surgical treatment for stress incontinence after prostate surgery with good long-term results in terms of continence and quality of life [4, 5]. However, besides patients' requirement of mental capacity and fine-motor control to operate the implanted pump, the significant reoperation rate ≥35% due to well-known complications such as cuff erosion, infection, or mechanical problems lead to an establishment of minimal invasive sling systems for treatment of urinary incontinence in the last years [6]. The retrourethral transobturator sling suspension (RTS), (AdVance male sling, American Medical Systems, Minnetonka, MN, USA), introduced in 2006, offers a functional approach by relocating the descent proximal urethra into the original anatomic position and thus allowing adequate function of the sphincter [7, 8]. Therefore sufficient sphincter contraction with or without complete closure is essential for the success of this approach. Promising results in recent studies show success rates from 54–80% regarding improved continence with few serious complications. [9–11]. However, failure rates of up to 36.5% and even worsening in 9% have been reported [12]. Taking these objections into account, this study was performed to prospectively evaluate the functional outcome of the RTS at our institution.

## 2. Patients and Methods

From September 2010 to September 2011, 32 patients with mild to severe stress urinary incontinence after prostate surgery were treated with RTS in the Department of Urology, University Hospital Ulm. The median age was 70.5 years (range: 61–88). Twenty-eight patients (87.5%) had undergone radical prostatectomy for prostate cancer, the remaining 4 patients (12.5%) had been treated with transurethral resection of the prostate. The median time between prostate surgery to sling implantation was 56 months (range, 6–161). The implantation of the male sling system was performed at least 6 months after initial treatment. The patient population comprised 10 men (31.3%) after additional pelvic radiotherapy. Ten patients (31.3%) were treated with transurethral incision of the bladder neck in case of bladder-neck obstruction prior (*n* = 8) to or during (*n* = 2) the implantation of the RTS. Six men (18.8%) had been treated with bulking agents without success before sling implantation. All patients had adequate prior treatment by pelvic floor muscle training. Preoperative work-up included physical examination, uroflowmetry (*Q*_max⁡_), postvoid residual urine (PVR) and flexible urethroscopy to assess sphincter function and mobility of the membranous urethra. The “repositioning test” [9, 13] was used to simulate the function of male sling before implantation. Only patients with sphincter contraction with or without complete sphincter closure received an RTS. ICIQ-UI SF 2004 score, a validated self-report questionnaire, was assessed to evaluate urinary incontinence and its impact on quality of life. Degree of incontinence was classified by the number of pads used per 24 hours and categorized in 3 grades (mild: 1-2, moderate: 3–5; severe: >5 pads/24 h).  Table 1 presents patient characteristics before sling implantation in detail. 

According to a prospective protocol, patients were reevaluated after 3, 6, and 12 months concerning the number of pads used daily, the current ICIQ-UI SF score, *Q*_max⁡_, and PVR. Cure was defined as no pad usage, improvement was defined as a use of 1-2 pads/day or ≥50% reduction of the preoperative pad use. Treatment was defined as success if patients were either cured or improved. All complications were recorded and assessed from review of the patients' clinical record charts. 

### 2.1. Surgical Treatment

The surgical implantation of male sling was performed as described before [7]. Intraoperative urethroscopy was performed in all cases to validate correct sling placement and to exclude urethral lesions. All sling implantations were performed by one surgeon (F.J.). 

### 2.2. Statistical Data Analysis

SPSS 17.0 software was used for statistical analysis. Quantitative values were compared using the Mann-Whitney *U*-test. Chi-square tests were performed to compare the success and failure groups by demographic, clinical, surgical, and follow-up parameters. An error probability of *P* < 0.05 was defined as the significance limit.

## 3. Results

The median followup was 9 months (range, 3–14). Based on pad test results at last followup the cure rate (no pad usage) was 56.3% (18 of 32 patients). The improvement rate (1-2 pads/day or ≥50% reduction) was 21.9% (7 patients). No improvement was seen in 21.9% (7 patients). The success rate was durable since only one patient initially classified as cured at the 3-month visit subsequently had to use pads again in the course of followup. Patients with severe SUI (*n* = 8) showed a trend of inferior cure rates compared to patients with mild (*n* = 6) and moderate (*n* = 18) SUI; however, this was not significant (25% versus 83.3% and 61.1%, *P* = 0.075, Fisher's exact test) (see Table 2). Overall mean ± SD pad use decreased from 5.1 ± 2.8 (median: 4, range: 2–10) to 1.8 ± 2.7 (median: 0, range: 0–10) pads daily (*P* < 0.001, *U* test). The ICIQ-UI SF score improved from a mean of 15.4 ± 3.5 (median: 16, range: 7–21) before sling implantation to 5.7 ± 6.3 (median: 4, range: 0–18) after surgery (*P* < 0.001; *U* test) (see Table 3). Twenty-one patients (65.2%) demonstrated a 50% reduction or more of the ICIQ-UI SF score after sling implantation. The 50% decrease of daily pad use was significantly associated with the 50% decrease of ICIQ-IU SF score indicating a strong correlation between both parameters (*P* < 0.001, Fisher's exact test). 

To evaluate the potential impact of prior treatment on the sphincter region and thus success of the RTS implantation several subgroup analysis was performed. 

Patients with prior pelvic radiotherapy (*n* = 10, 31.3%) demonstrated a cure rate of 30% (*n* = 3/10) and an improvement rate of 30% (*n* = 3/10), respectively. The failure rate in patients with prior radiotherapy was 40% (*n* = 4/10). The ICIQ-UI SF score was decreased by ≥50% in 40% of these patients. However, there was no statistical difference regarding pad use and ICIQ-UI SF score compared to patients without prior radiotherapy (≥50% pad reduction 60.0% versus 81.8%, *P* = 0.218 and ≥50% ICIQ-UI SF score decrease. 44.4% versus 77.3% *P* = 0.105, respectively; without prior radiotherapy's exact test). 

Considering patients with previous or simultaneous transurethral incision of the bladder neck (*n* = 10, 31.3%) the cure rate was 30%, the improvement rate 20%, and the failure rate 50%. Again, there was a trend towards worse outcome regarding decrease of daily pad use compared to patients without transurethral incision (≥50%; 50.0% versus 86.8%; *P* = 0.072, Fisher's exact test). However, the reduction of ICIQ-UI SF score demonstrated a significantly worse outcome in these patients (≥50%; 33.3% versus 81.8%; *P* = 0.015, Fisher's exact test). Neither pretreatment with bulking agents nor patient age (≥70 yrs. versus <70 yrs.) had impact on improvement of pad use or ICIQ-UI SF score after sling implantation. 

No changes in postvoid residual urine (PVR) were observed after the implantation of the RTS (mean: 7.3 ± 12.8 mL versus 11.0 ± 19.0 mL; *P* = 0.381, *U* test). Uroflowmetry demonstrated significant decreased *Q*_max⁡_ rates (mean: 20.3 ± 11.9 versus 23.9 ± 13.6 mL/sec) after sling implantation (*P* < 0.001, *U* test). Table 3 summarizes these events. 

### 3.1. Adverse Events

No perioperative serious complications occurred. In one patient the RTS had to be removed because of sling dislocation and persistent perineal pain. The patient had undergone prior pelvic radiotherapy and palliative transurethral laser resection before sling implantation. Postoperative acute urinary retention (AUR) was seen in five patients (15.6%). All of these patients were treated with a transurethral catheter. In all cases the catheter could be removed after 1–3 weeks without further treatment with residual urine ≤50 mL at time of catheter removal. 3 patients (9.3%) had mild local wound infection/inflammation and were treated conservatively with oral antibiotics. 

## 4. Discussion

Several reports described promising results of RTS implantation on postprostatectomy urinary stress incontinence. [7, 9, 10, 13, 14]. Midterm outcome in a recent study showed sustainable results over a followup period of up to two years [15]. The cure rate ranged between 51–73% in previous studies [9–11, 16–18]. Most common complications of the male sling are urinary retention, impaired healing, and sling dislocation due to inflammation or misplacement [13, 19]. In our present study the cure rate was 56.3%. Moreover, in 21.9% of the patients the incontinence was significantly improved. Considering these data, implantation of RTS is a valid procedure for treatment of postprostate-surgery incontinence. The significant decrease of the ICIQ-UI SF score from a median of 15.4 before sling implantation to 5.7 after surgery underscores this observation. Most common complications were acute urinary retention in 15.6% and wound healing disorders in 9.3%. Both occurrences were treated conservatively. One patient underwent sling removal because of dislocation of the sling system and persistent perineal pain. While acute urinary retention was a transient postoperative observation, mild reduction of *Q*_max⁡_ was a common finding after male sling implantation in our study suggesting that the implantation and function of the RTS result at least partly in obstruction. In contrast to our observation urodynamic evaluation performed in previous studies had not revealed significant changes of maximum flow rates following sling implantation [15, 16]. However, in the present study the clinical impact of the reduced *Q*_max⁡_ could almost be disregarded. Residual urine volume demonstrated no significant change after sling implantation and urinary frequency seemed to be unaffected. 

Most accurate means of predicting treatment success and subsequent selection of appropriate patients for functional transobturator sling implantation are still under debate. 

In the present study severity of incontinence showed a trend to negative impact on the cure rate. This observation was found to be significant in a few studies [10, 11, 20, 21], whereas no correlation was found in other reports [9, 12]. Even though the cure rate was only 25% in patients with severe SUI, the present success rate (cure rate + improvement rate) of 62.5% in this challenging cohort demonstrates that patients with severe incontinence can benefit from sling implantation. Nevertheless, precise patient information about the decreased cure rate is needed if male sling implantation is the chosen treatment in these patients. 

Although our results are hampered by the small number of cases, analysis of patients with history of radiation therapy showed a tendency of worse outcome in terms of pad use and ICIQ-UI SF score, respectively. This observation is in line with Cornu et al. who noticed in patients with prior radiotherapy (*n* = 17) a success rate of 59% (9 cured, 1 improved) compared to 85% in patients without history of pelvic radiation indicating that prior radiation could predict inferior clinical outcome [10]. In a prospective study Bauer et al. reported similar results with a success rate of 50% (25% cure rate, 25% improvement rate) in selected patients (*n* = 24) after radical prostatectomy and adjuvant radiotherapy [22]. One explanation for the worse outcome in these patients might be a direct radiogenic damage of the intrinsic sphincter in combination with an increasing immobility of the urethra caused by the irradiated tissue in the perineal area [22]. 

Moreover, patients with previous or simultaneous anastomotic stricture surgery (*n* = 10) showed a trend towards inferior outcome regarding decrease of daily pad use compared to patients without history of urethral surgery (50.0% versus 86.8%). Additionally, the ICIQ-UI SF score demonstrated a significant worse outcome in these patients (≥50% reduction; 33.3% versus 81.8%). This corresponds to midterm results reported by Cornu et al. on a subgroup of 18 patients with a history of surgery for urethral stenosis demonstrating a significant association with persistent pad use after sling implantation [20]. The lower success rate in patients with prior transurethral surgery may be caused by the immobility of a “stiff” low compliant proximal urethra resulting from intrinsic scar tissue. Our study indicates that prior therapy with potentially negative impact on the urethral mobility has a negative influence on treatment success. Therefore, in this challenging cohort we recommend using the functional treatment approach of the RTS very cautiously. 

The limitation of our study is certainly the low number of patients with a mean followup of 9 months after implantation. Long-term prospective studies with focus on risk factors of treatment failure after RTS implantation are warranted to prove real value. 

## 5. Conclusions

The retrourethral transobturator male sling represents a safe and effective treatment option for patients with postprostate-surgery SUI. History of radiotherapy and urethral surgery as well as severe incontinence should be carefully considered in the preoperative selection of patients since in this subgroup the success rate of AdVance male sling implantation could be lower. 

## Figures and Tables

**Table 1:** Patient characteristics before sling implantation.

Variable	Value
Age at sling implantation, years	
Median (range)	70.5 (61–88)
Prostatectomy *n* (%)	
Retropubic	22 (68)
Laparoscopic	3 (9.4)
Perineal	3 (9.4)
TUR-P	6 (12.5)
Pretreatment *n* (%)	
Radiotherapy	10 (31.3)
Bladder neck incision	10 (31.3)
Bulking agents	6 (12.5)
Daily pad use, pads/day	
Median (range)	4.0 (2–10)
Mean	5.1
Grade of incontinence, no of patients (%)	
Mild: 1-2 pads/day	6
Moderate: 3–5 pads/day	18
Severe: ≥6 pads/day	8

**Table 2:** Postoperative results at maximum followup.

	All patients	Patients with mild incontinence (*n*)	Patients with moderate incontinence (*n*)	Patients with severe incontinence (*n*)
	*N* = 32	*N* = 6	*N* = 18	*N* = 8
Success rate^†^	78.2% (25)	83.3% (5)	83.3% (15)	62.5% (5)
Cure rate	56.3% (18)	83.3% (5)	61.1% (11)	25.0% (2)
Improved rate	21.9% (7)	—	22.2% (4)	37.5% (3)
Failed	21.9% (7)	16.7% (1)	16.6% (3)	37.5% (3)

^†^success rate = cure rate + improved rate.

**Table 3:** Results of RTS treatment at maximum followup.

Variable	Before sling implantation	After sling implantation
Daily pad use, pads/day		
Median (range)	4 (2–10)	0 (0–10)^†^
Mean	5.06	1.75
ICIQ-UI SF score		
Median (range)	16 (7–21)	4 (0–18)^‡^
Mean	15.39	5.65
PVR*, mL		
Median (range)	0 (0–40)	0 (0–70)^§^
Mean	7.33	11.00
Maximum uroflowmetry, mL/s		
Median (range)	21.9 (2.3–53.6)	17.2 (5.9–43.4)^¶^
Mean	23.97	20.33

*postvoid residual urine

^†^ *U*-Test; *P* < 0.001

^‡^ *U*-Test; *P* < 0.001

^§^ *U*-Test; *P* = 0.381

^¶^ *U*-Test; *P* < 0.001.
